# Dehydroabietic Acid Derivative QC2 Induces Oncosis in Hepatocellular Carcinoma Cells

**DOI:** 10.1155/2014/682197

**Published:** 2014-07-06

**Authors:** Guang Zhang, Chunping Jiang, Zhongxia Wang, Weibo Chen, Wen Gu, Yitao Ding

**Affiliations:** ^1^Department of Hepatobiliary Surgery, Drum Tower Clinical Medical College of Nanjing Medical University, Nanjing, Jiangsu 210008, China; ^2^Department of Hepatobiliary Surgery, Affiliated Drum Tower Hospital of Nanjing University Medical School, Nanjing, Jiangsu 210008, China; ^3^School of Medicine, Nanjing University, Nanjing, Jiangsu 210093, China; ^4^College of Chemical Engineering, Nanjing Forestry University, Nanjing, Jiangsu 210037, China

## Abstract

*Aim.* Rosin, the traditional Chinese medicine, is reported to be able to inhibit skin cancer cell lines. In this report, we investigate the inhibitory effect against HCC cells of QC2, the derivative of rosin's main components dehydroabietic acid. *Methods.* MTT assay was used to determine the cytotoxicity of QC2. Morphological changes were observed by time-lapse microscopy and transmission electron microscopy and the cytoskeleton changes were observed by laser-scanning confocal microscopy. Cytomembrane integrity and organelles damage were confirmed by detection of the reactive oxygen (ROS), lactate dehydrogenase (LDH), and mitochondrial membrane potential (Δ*ψ*m). The underlying mechanism was manifested by Western blotting. The oncotic cell death was further confirmed by detection of oncosis related protein calpain. *Results.* Swelling cell type and destroyed cytoskeleton were observed in QC2-treated HCC cells. Organelle damage was visualized by transmission electron microscopy. The detection of ROS accumulation, increased LDH release, and decreased ATP and Δ*ψ*m confirmed the cell death. The oncotic related protein calpain was found to increase time-dependently in QC2-treated HCC cells, while its inhibitor PD150606 attenuated the cytotoxicity. *Conclusions.* Dehydroabietic acid derivative QC2 activated oncosis related protein calpain to induce the damage of cytomembrane and organelles which finally lead to oncosis in HCC cells.

## 1. Introduction

HCC, the fifth common cancer, causes the third most cancer-related death worldwide [1]. Although therapy strategies have been developed, the outcome of this fatal disease is unsatisfactory, with the five-year overall survival rate being less than 11% [2]. Various reasons might contribute to this dilemma situation among which the bad behavior of HCC might be the most important one. The general therapies for HCC are comprised of surgery and palliative treatments. Hepatectomy and liver transplantation remain the curative treatments for this fatal disease while only 30–40% patients are eligible [3]. For those end-stage patients, noncurative strategies including transcatheter arterial chemoembolization (TACE) and radiofrequency ablation (RFA) are available but only few-month survival time is prolonged [3]. Unlike other cancers, specific molecular targets in HCC are deficient which results in lacking of preferable molecular targeted drugs except for sorafenib [4, 5]. On the other hand, the overexpression of multidrug-resistance gene leads to the insensitivity of systemic chemotherapy [6]. As a result, it is urgent and significant to develop novel therapies for HCC.

Natural derived compounds and their derivatives have been developed into anticancer agents for decades. Newman et al. [7] analyzed 92 commercially available anticancer drugs. Of all the drugs, they found that 62% were related to the natural products. Natural compounds and their derivatives play a critical role in the discovery of new anticancer agents which can be proved by the approval of commonly used drugs in clinic. These agents include toposide (established from the podophyllin), paclitaxel (originated from the rind of* Taxus brevifolia*), and doxorubicin (extracted from the streptomycin). Rosin, the natural product of pine tree, is widely used in traditional Chinese medicine. Tanaka tested the anticancer effects of the main components of rosin and found that 4 out of 8 compounds were toxic to the two skin cancer cell lines [8]. Gu et al. did chemical modification to the dehydroabietic acid, an effective component of rosin, according to Tanaka's research and tested the antibacterial activity [9]. The newly synthesized compounds showed potential inhibitory activity to different bacteria. Nevertheless, the anticancer effect remained unknown.

In this study, we used an N-substituted 1H-dibenzo[a,c]carbazole derivative from dehydroabietic acid, which was granted by Gu, and focused on the anticancer effects and the underlying mechanisms.

## 2. Methods

### 2.1. Synthesis of Dehydroabietic Acid Derivative QC2

**Scheme 1:**
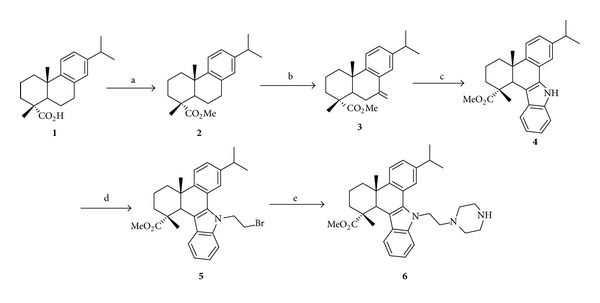
Synthetic route of compound QC2 (**6**) from dehydroabietic acid (**1**). Reagents and conditions: (a) (i) SOCl_2_, benzene, reflux, and 3 h; (ii) MeOH, reflux, and 2 h; (b) CrO_3_, AcOH, Ac_2_O, 0°C to rt, and 12 h; (c) phenylhydrazine hydrochloride, EtOH, conc. HCl, reflux, and 3 h; (d) 1,2-dibromoethane, TBAB, NaOH, benzene, rt, and 12 h; (e) piperazine, K_2_CO_3_, KI, MeCN, reflux, and 8 h.

**Figure 1:**
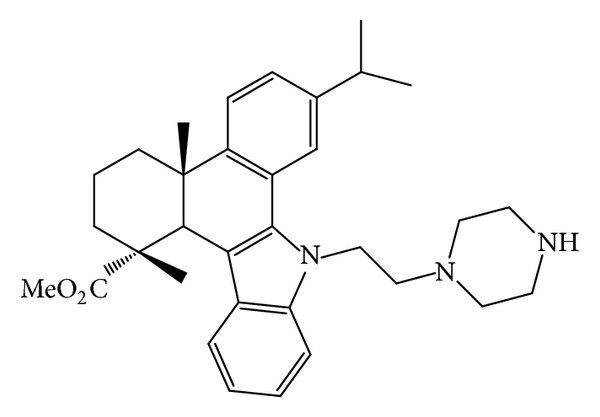
Structure of QC2 (2,3,4,4a,9,13c-hexahydro-7-isopropyl-1,4a-dimethyl-9-(2-(piperazin-1-yl)ethyl)-1H-dibenzo[a,c]carbazole-1-carboxylic acid methyl ester).

QC2 (Figure 1) is an N-substituted 1H-dibenzo[a,c]carbazole derivative synthesized from dehydroabietic acid. The synthetic route of compound QC2 is illustrated in Scheme 1 and the detailed methods could be found in the paper of Gu and coworkers [10].

### 2.2. Cell Culture and Reagents

Human HCC cell lines Hep3B, HepG2, and Huh7 were obtained from the Cell Bank of Chinese Academy of Sciences (Shanghai, China); SMMC-7721 and human hepatocyte LO2 cells were purchased from Cell Bank of Xiangya Central Experiment Laboratory of Central South University (Changsha, China). The cells were maintained in Dulbecco's modified Eagle's medium (DMEM) (GIBCO BRL, Gaithersburg, MD) containing 10% fetal bovine serum (FBS) (GIBCO BRL, Gaithersburg, MD), 100 U/mL penicillin, and 100 *μ*g/mL streptomycin. The cells were cultured in 5% CO_2_ at 37°C. Purified QC2 was dissolved in dimethylsulfoxide (DMSO) at a concentration of 10 mg/mL and stocked at −20°C. 3-(4,5-dimethylthiazol)-2,5-diphenyltetrazolium (MTT) and anti-tubulin-Cy3 antibody were purchased from Sigma Aldrich (St. Louis, MO). Alexa Fluor 594 phalloidin and 4′,6-diamidino-2-phenylindole (DAPI) were purchased from Life Technologies (Invitrogen, Carlsbad, CA). Calpain, caspase-3, actin, and tubulin antibody were obtained from Cell Signaling Technology (Danvers, MA, USA).

### 2.3. MTT Assay

Cytotoxicity was determined by MTT assay. Generally, 5 × 10^3^ cells were seeded into 96-well plates and cultured for 24 hours. After being treated with indicated reagents for different times, cells were incubated with 20 *μ*L MTT (5 mg/mL) for another 4 hours. Then, the culture medium was discarded and 150 *μ*L DMSO was added to dissolve the MTT formazan precipitates. The optical density was measured using a microplate reader (Molecular Devices, Sunnyvale, CA) at 490 nm. The cytotoxicity of QC2 to each cell line was determined by the preceding results.

### 2.4. Annexin V-FITC/PI Staining

Cells were collected after being treated with different concentration of QC2. The cell aggregates were resuspended with the binding buffer and incubated with Annexin V-FITC for 30 minutes at room temperature. The supernatants were discarded after centrifugation. Cells were resuspended with the binding buffer and incubated with PI and subjected to flow cytometry analysis.

### 2.5. Western Blotting

To determine the level of indicated proteins, QC2-treated cell lysates were prepared as described [11]. Twenty *μ*g proteins was analyzed by Western blot as described. The PVDF membranes with transferred proteins were incubated with primary antibodies at 4°C overnight and HRP-conjugated secondary antibodies at room temperature for 2 hours. The signal was developed by the enhanced chemiluminescence (ECL) reagent (Millipore, Bedford, MA) and visualized by FlourChem FC2 Imaging System (Alpha Innotech, San Leandro, CA).

### 2.6. Transmission Electron Microscopy

Cells treated with QC2 were digested by trypsin and then centrifuged at 1000 r/min. Thereafter, cells were fixed with 4% glutaraldehyde and postfixed with 0.15 mol/L phosphate buffer with 3% OsO_4_ both for 1 hour. Dehydration was carried out in alcohol-water solutions of varying concentrations and 100% propylene oxide. Then, cells were embedded by a mixture of propylene oxide and araldite at the ratio of 1 : 3 (v/v) concentrations overnight after been embedded at 1 : 1 for 1 hour. After treated with undiluted resin for 1 h, cells were polymerized at 60°C for 3-4 days. Ultrathin sections were obtained and stained with 1% toluidine blue. Ultrastructure was observed by a transmission electron microscope (JEM-1010, Japan).

### 2.7. Fluorescence Microscopy

Cells were seeded on the cover slides in 24-well plates. Fixation was performed with 4% paraformaldehyde for 10 minutes and permeabilization was carried out with 0.1% Triton X-100 for 5 minutes. After washing for 5 times, cells were preincubated with PBS containing 1% BSA for 30 minutes. Staining solution was added after discarding the 1% BSA. Incubation time was according to the manufacturer's instructions followed by washing with PBS for 5 times. When nuclear staining was required, DAPI or PI was used afterwards. Cover slides were mounted onto glass slides and observed by a fluorescence microscope.

### 2.8. Lactate Dehydrogenase (LDH) Release Assay

5 × 10^3^ cells were seeded into 96-well plates. After being treated with 5 mg/mL QC2 for indicated time, the plates were centrifuged to get the supernatants. The LDH level of the supernatants was detected by a LDH assay kit (Beyotime, Nantong, China). The optical density was measured using a microplate reader (Molecular Devices, Sunnyvale, CA) at 450 nm.

### 2.9. Detection of Intracellular ATP

As a parameter of Na^+^, K^+^-ATPase activity, the intracellular ATP was detected. We carried out this detection using a commercial detection kit (Beyotime, Nantong, China) according to the manufacturer's instructions. Luciferase activity was measured as the instruction of ATP level by Dual-Luciferase Reporter Assay System.

### 2.10. Mitochondrial Membrane Potential Measurement

Changes of mitochondrial membrane potential were the earliest indication of cell death but not the distinction between apoptosis and oncosis. We measured the mitochondrial membrane potential using a commercial kit (Beyotime, Nantong, China) according to the manufacturer's protocols. Cells were stained by the kit and fluorescence intensities were detected by flow cytometry. The Δ*ψ*m was calculated through the fluorescence intensities using the Cell Quest software (Becton Dickinson, Bedford, MA).

### 2.11. Reactive Oxygen Species Assay

Intracellular ROS level was measured using the 2′,7′-dichlorofluorescein diacetate (DCFH-DA). Pretreated cells were reacted with DCFH-DA according to the manufacture's protocol. Cellular fluorescence was measured by flow cytometry.

### 2.12. Statistical Analysis

Numeric data were shown as means ± SD. Statistical significance between two groups was analyzed by two-way ANOVA. *P* < 0.05 was considered statistically significant.

## 3. Results

### 3.1. QC2 Exerted Cytotoxicity against HCC Cell Lines as well as Human Hepatocytes

**Figure 2:**
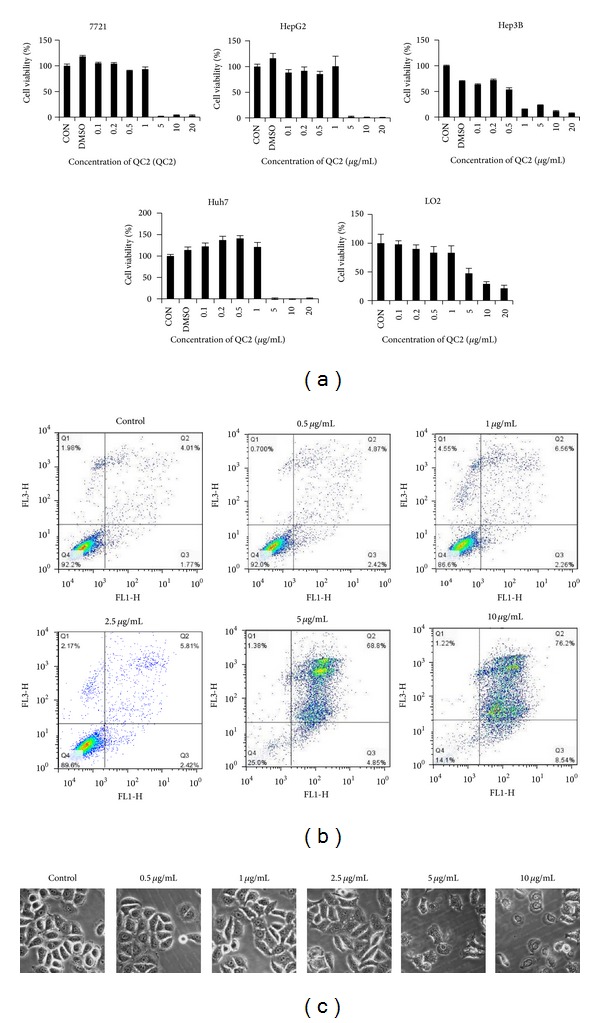
QC2 induced cell death in HCC and human hepatocytes. (a) QC2 exerted cytotoxicity in four HCC cell lines as well as in human hepatocytes; the IC_50_ values were 0.37 *μ*g/mL, 1.17 *μ*g/mL, 3 *μ*g/mL, 2.67 *μ*g/mL, and 4.22 *μ*g/mL, respectively. (b) QC2 induced cell death in SMMC-7721 cells was testified by flow cytometry. (c) QC2 dose-dependently induced morphological changes in SMMC-7721 cells.

We studied the cytotoxicity of QC2 on SMMC-7721, Hep3B, HepG2, and Huh7 by MTT assay and flow cytometry (Figures 2(a) and 2(b)). Of all the four HCC cell lines, QC2 showed high cytotoxicity that 5 *μ*g/mL QC2 was lethal to most HCC cells. The IC_50_ values of four HCC cell lines were 0.37 *μ*g/mL for Hep3B cells, 1.17 *μ*g/mL for HepG2 cells, 3 *μ*g/mL for Huh7 cells, and 2.67 *μ*g/mL for SMMC-7721 cells, respectively. The IC_50_ value for LO2 cells was higher: 4.22 *μ*g/mL. Something interesting was that Huh7 cells and SMCC-7721 died at the concentration of 5 *μ*g/mL while nearly all cells survived at 2.5 *μ*g/mL. During the cytotoxicity determination, we also observed the morphologic change. Time-lapse microscopy showed that cells membrane became incomplete and cells swelling happened after plasma membrane blebbing appeared (Figure 2(c)).

### 3.2. QC2 Ravaged the Integrity of Cell Membrane

**Figure 3:**
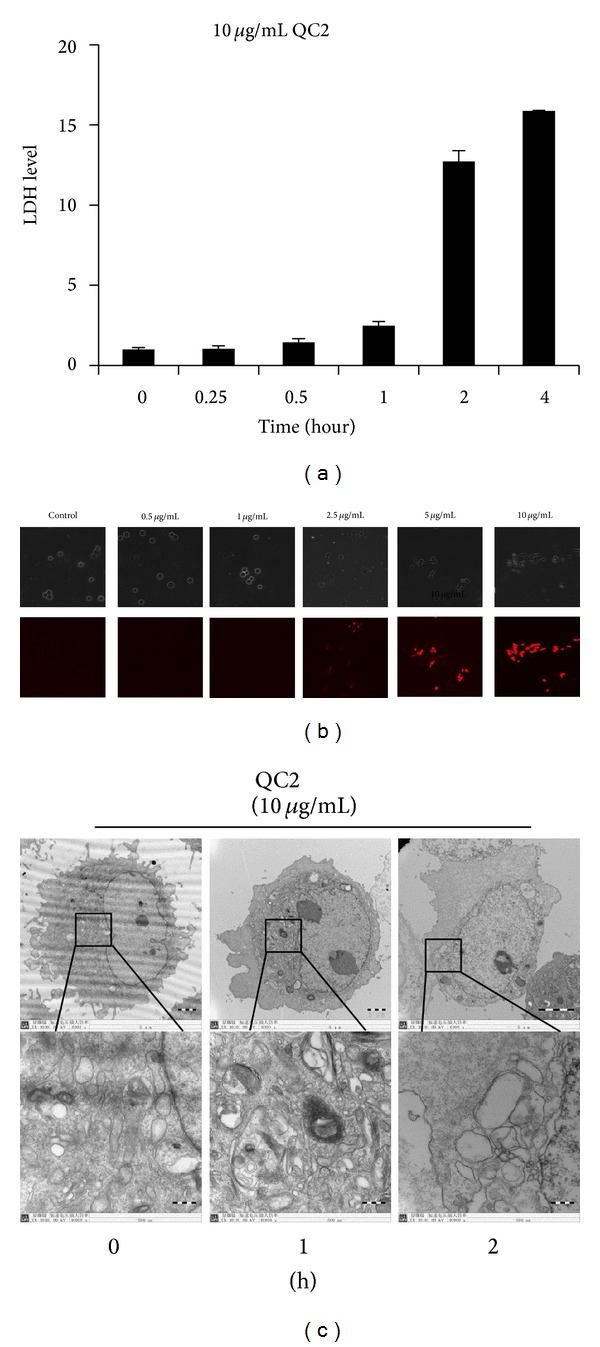
QC2 induced destruction of cytomembrane and ultrastructural changes in SMMC-7721 cells. (a) LDH level time-dependently increased in the supernatants of QC2-treated cells. (b) PI uptake of SMMC-7721 cells accumulated along with increase of QC2 concentration. (c) Electron micrographs of QC2-treated SMMC-7721 cells showed ultrastructure changes.

LDH would be released into the medium and the nucleus would become easily stained when the cell membrane is destructed. Hence, we evaluated the integrity of cell membrane by LDH release assay and PI uptake. We treated cells with 10 *μ*g/mL QC2 from 0.25 hour to 4 hours. As shown in Figure 3(a), LDH level stayed low during the first 1 hour and increased at 2 hours which suggested a membrane rupture. The dose-dependent PI uptake was in accordance with the LDH release assay. As shown in Figure 3(b), cells retained smooth morphology and no fluorescence was seen in the control group or the low dose groups while cells became swelled and red fluorescence stained by PI in the groups over 2.5 *μ*g/mL.

### 3.3. Ultrastructure Examination Confirmed Oncotic Cell Death

Morphology changes were the direct distinction between apoptosis and oncosis. Due to the extraordinary morphology changes observed by time-lapse microscopy, we examined the ultrastructure of QC2-treated cells (Figure 3(c)). After being treated with QC2 for 1 hour, pseudopodia disappeared because of cell swelling and organelles injured apparently which might lead to membrane blebbing. Nucleus remained integrated but dilated as well as the whole cell. Two hours later, plasma membrane got destroyed and organelles became fuzzy. Chromosomes condensation was observed in the 2-hour group. The above ultrastructure changes confirmed our hypothesis that QC2 induced oncosis in HCC cells.

### 3.4. Cytoskeleton Was Destroyed by QC2

**Figure 4:**
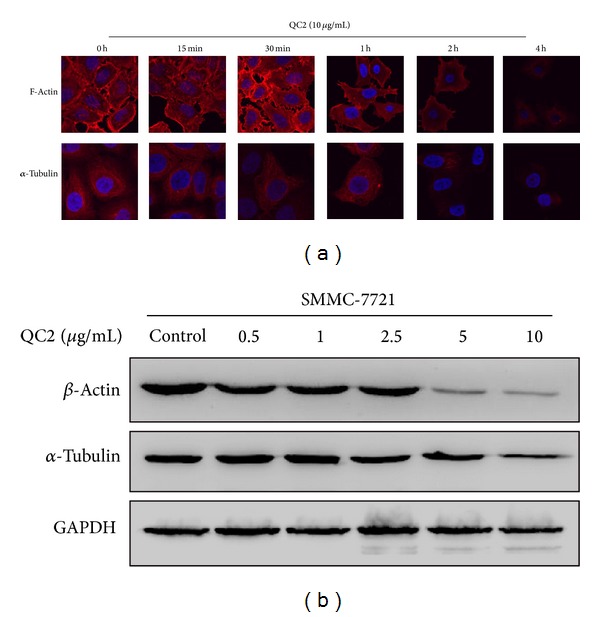
QC2 destroyed the cytoskeleton of SMMC-7721 cells. (a) Immunostaining of cytoskeleton proteins actin and tubulin. (b) Expression of actin and tubulin in SMMC-7721 cells treated with or without QC2; GAPDH was set as internal standard to normalize loadings.

As ultrastructure examination showed fuzzy morphology at the end of QC2 treatment, we speculated that the cytoskeleton might also be affected. So we testified the cytoskeleton changes by fluorescence microscopy and Western blotting. As shown in Figure 4(a), the staining of microtubules and microfilaments weakened with the extended response time. Actin and tubulin protein were also detected and both suffered a significant decrease (Figure 4(b)).

### 3.5. QC2 Induced Oncosis Was Not Reversed by Caspase-3 Inhibitor z-VAD-fmk or Necrosis Inhibitor Necrostatin

**Figure 5:**
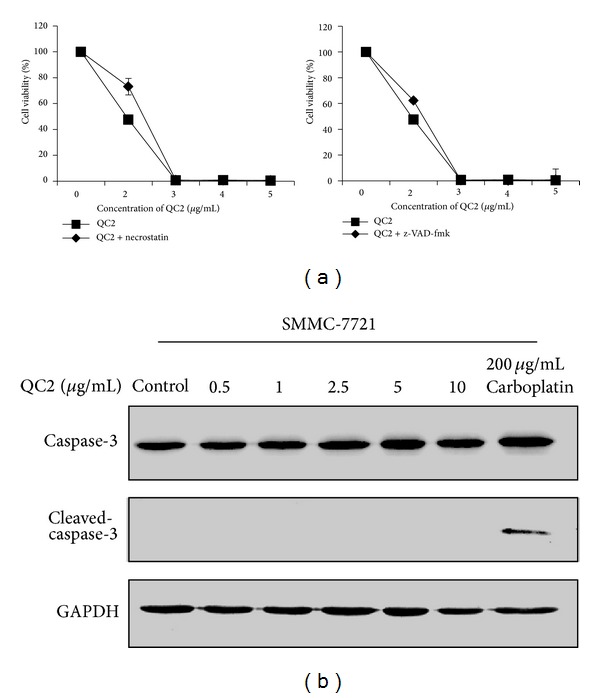
QC2 induced oncosis was not interfered by neither apoptosis inhibitor nor necrosis inhibitor and no caspase-3 activation was detected under QC2 treatment. (a) No significant change of cell viability was detected when SMMC-7721 cells were pretreated with or without z-VAD-FMK or necrostatin (*P* > 0.05). (b) As QC2 concentration increased, no cleaved-caspase-3 was detected while it appeared in 200 *μ*g/mL carboplatin treated cells.

As an executioner caspase, caspase-3 plays a crucial role both in extrinsic (death ligand) and intrinsic (mitochondrial) apoptosis pathways [12, 13]. Blockage of caspase-3 by its inhibitor z-VAD-fmk could abolish the apoptotic events; the same thing would happen in necrosis by necrostatin [14]. To further distinguish oncosis from apoptosis and necrosis, we pretreated cells with z-VAD-fmk (50*μ*M) or necrostatin (500 nM) before QC2 treatment. Results came out that no statistical difference was detected when using z-VAD-fmk or necrostatin (Figures 5(a) and 5(b)). During the protein detection, carboplatin was set as the positive control for activated caspase-3 detection but no such activated caspase-3 was found in the QC2 groups (Figure 5(c)). All these results suggested that the oncosis induced by QC2 in SMMC-7721 cells was different from apoptosis and necrosis.

### 3.6. QC2 Caused ATP Depletion, ROS Generation, and Mitochondrial Membrane Potential Changes in HCC Cells

**Figure 6:**
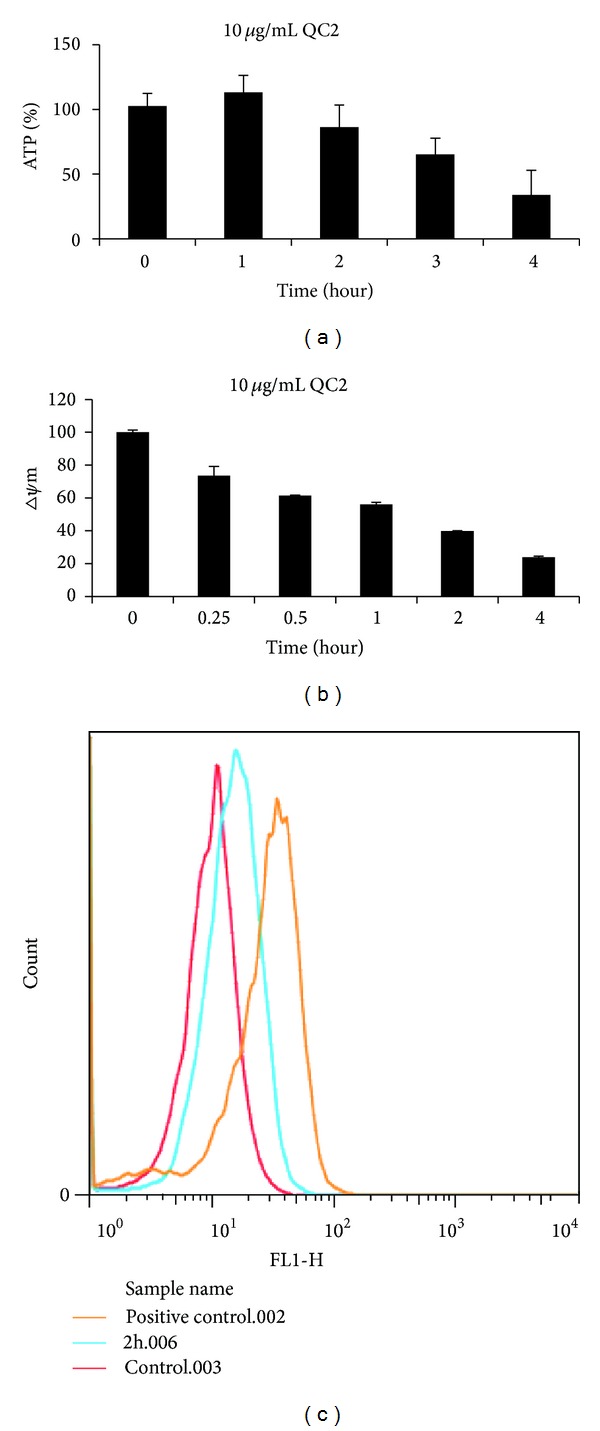
ATP deletion, Δ*ψ*m collapse, and ROS generation were observed in oncotic cells. ((a) and (b)) ATP and Δ*ψ*m level generally decreased in 10 *μ*g/mL QC2-treated SMMC-7721 cells time-dependently. (c) ROS accumulation was detected in QC2-treated cells.

Researchers have found that oncosis started with ATP depletion; then, ion pumps were influenced and Δ*ψ*m collapsed [15]. As an indicator of mitochondria damage, ROS was also detected. ATP decreased time-dependently as well as Δ*ψ*m (Figures 6(a) and 6(b)). Compared to the positive control, 5 mg/mL Rosup, the QC2-treated cells showed a similar tendency of ROS accumulation (Figure 6(c)). ROS generation and mitochondrial membrane changes were also key events in apoptosis and necrosis; however, ATP depletion emerged only in oncosis. The ATP depletion detected in our research confirmed QC2 induced oncosis in HCC cells.

### 3.7. Oncosis Related Protein Calpain Activated in QC2-Treated HCC Cells

**Figure 7:**
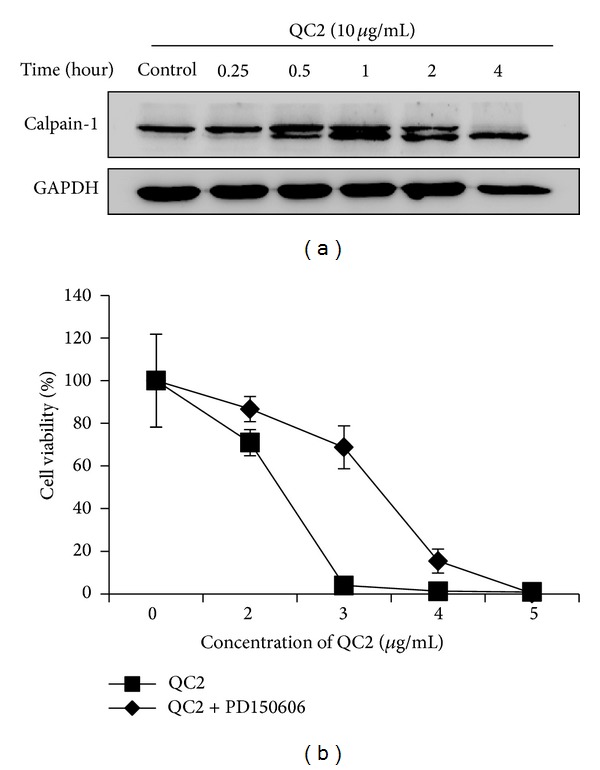
Calpain-1 increased during oncosis and its inhibition attenuated the cytotoxicity of QC2. (a) Calpain-1 increased and gradually autolyzed into 76 kDa fragments under QC2 treatment. (b) Significant change of cell viability was observed in SMMC-7721 cells when pretreated with the calpain inhibitor PD150606 (*P* < 0.05).

Various studies have found that calpain, a kind of calcium-dependent thiol proteinase, mediated the oncotic event in different cells. Researchers have found that calpain increased before membrane damage and inhibition of calpain could protect cells from cell death [15], but whether calpain-1 or calpain-2 was involved remained unknown. In our study, we confirmed the activation of calpain-1 in QC2-treated cells by detecting the level of this protein. As shown in Figure 7(a), calpain-1 increased and the protein autolyzed from the molecular weight of 80 kDa to 76 kDa when treated with QC2. The increased calpain-1 level might imply the activation of this protein during the oncotic cell death. In the subsequent experiment, the calpain inhibitor PD150606 was adopted. To our delight, 100 *μ*M PD150606 partially abrogated the cytotoxicity of QC2 at the concentration of 3 *μ*g/mL (Figure 7(b)).

## 4. Discussion

HCC is one of the tumors with poor prognosis. The dilemma of HCC might lie in deficiency of effective drugs. According to the BCLC staging system, patients with advanced stage tumors are recommended to receive palliative treatments such as TACE, sorafenib, and symptomatic treatment [3]. Sorafenib is the only molecular-targeted drug that has been proved to have survival benefit for HCC patients although it can only prolong the median survival time for few months. Systemic chemotherapy has been proved to be useless for HCC but TACE, a kind of local chemotherapy, partially improves the survival time [16, 17]. During TACE, tumor tissues are perfused with chemotherapeutics like doxorubicin and cisplatin and the tumor supplying blood vessels are embolized. As a result, tumor tissues are suffering from ischemia and chemotherapeutics that HCC cells die of both apoptosis and necrosis. This therapeutic tragedy could suppress the tumor growth. However, it could not thoroughly remove the tumor cells that most patients would experience the tumor recurrence. Neovascularization and resistance to chemotherapeutics might be the reason [11]. Thus, more effective chemotherapeutics need to be discovered.

Cell death is a topic which has been debated for decades. Therapeutic drugs are also developed based on their cytotoxicity. In the past, apoptosis and accidental cell death are the main cell death types that have been widely studied [18]. Apoptosis is a programed process while accidental cell death is unpredictable [19]. Autophagic cell death is a recently discovered cell death type [20]. Of all these types of cell death, apoptosis is the most widely studied and its molecular mechanisms are well revealed. Apoptosis starts with damage of nuclear chromatin; then the cytoplasm condensates and DNA degrades, and at last cell membrane blebs and the whole cell fragmentates into apoptotic bodies [18]. Oncosis, which is originated from the word “swell,” is one type of accidental cell death and is earlier known for being common in ischemia issues. During the process of oncosis, cells suffer from the deletion of intracellular ATP caused by ischemia or other stuff; then, cytomembrane becomes unstable and cellular swelling happens [21]. As a result, the morphological changes of oncosis turn out to be different: the incompleteness of cytomembrane, blebbing of plasma membrane, clumping of nuclear chromatin, and dilation of endoplasmic reticulum (ER) [19]. Researchers also found that oncosis was accompanied with rearrangement of cytoskeleton [22]. Meanwhile, these two processes did not distinguish completely between each other; for example, the mitochondrial membrane potential changes and ROS accumulates and they both share the same ending, necrosis.

Despite the fact that the procedure of oncosis has been known for decades, the mechanisms were not fully understood. ATP deletion is thought to be the origin of this passive death type. Lieberthal et al. [23] used antimycin and 2-deoxyglucose to reduce ATP in mouse proximal tubular cells. They found that oncosis happened when 85% or more ATP was deleted but cells would survive or apoptosis would happen when less ATP was deleted. Liu et al. [15] found that the oncotic cell death could not be restored when the respiration and ion homeostasis were totally destroyed. In oncosis, the decrease in intracellular ATP (ATP[i]) resulted in the inactivation of Na^+^, K^+^-ATPase at the membrane; then, the intracellular Na^+^, Ca^2+^, and Cl^−^ arise along with water influx which leads to the cellular swelling. At the same time, the rapid increase of Ca^2+^[i] stimulates many signals and also results in the dysfunction of mitochondria after which the intracellular ATP synthesis is further influenced [24]. This positive feedback exacerbates cell death and the cytoskeleton also collapses as a result of Ca^2+^[i] increase.

Recently, oncosis regained researchers' attention as some agents exhibited anticancer activity via this process and certain novel anticancer agents were also established based on induction of oncotic cell death. Ma et al. [25] focused on an antibiotic isolated from* Pseudomonas jinjuensis*, which was previously proved to have anticancer potential, and found that this antibiotic could induce oncosis in breast cancer cells. Steroidal alkaloidal saponins were demonstrated to be natural anticancer agents as they induced apoptosis in cancer cells [26]. However, oncosis was also inducted by the solamargine, one of steroidal alkaloidal saponins [27]. Mechanisms were not mentioned in preceding studies. Artesunate was also a native compound which could induce oncosis in cancer cells. Zhou et al. [28] found that the increase of calpain-2 level was accompanied with the oncosis event induced by artesunate and they partly elucidated the mechanisms based on calpain-2 activation. The underlying mechanism of oncosis might be related to activation of calpain.

In our study, the QC2-treated cells showed a significant ATP decreasing level, while the ATP depletion was considered to be the initiation of oncosis [15]. The cytomembrane was destroyed due to the inactivation of ion pumps caused by ATP depletion. At the same time, ROS accumulated and Δ*ψ*m collapsed as the marker of cellular damage. Oncotic cell death was irreversible when mitochondrial function and ion homeostasis were totally destroyed [29]. Calpain activation also played a key role in this process but its autolysis was controversial [30]. For example, Molinari et al. [31] reported that calpain was activated at 80 kDa form but Kulkarni et al. [32] thought that the activation was accompanied with the autolysis. We studied the protein level of calpain-1 and found that its upregulation was accompanied with its autolysis as well as the oncotic events while its inhibition partially rescued the cells. As a calcium-activated neutral protease, calpain could hydrolyze many substrates including cytoskeletal proteins. The morphological changes and the destruction of cytoskeletal proteins might be related to the activation of calpain. Nevertheless, relationships between calpain and oncosis should be further illuminated.

Liver dysfunction is always accompanied with HCC [33]. The diseased liver may not sustain the cytotoxicity of chemotherapeutic agents. The cytotoxicity of QC2 to normal cells was also considered. We testified the sensitivity with a normal liver cell line LO2. The IC_50_ value for LO2 was higher than for HCC cell lines; however, the cytotoxicity should not be ignored. Further study on decreasing cytotoxicity against normal hepatocytes in vivo should be carried out.

In conclusion, QC2 showed a potential anticancer effect in HCC cell lines through inducing oncotic cell death. It might provide a novel therapeutic strategy for HCC and the safety needs to be further studied.
